# Methods for Elemental Analysis of Size-Resolved PM Samples Collected on Aluminium Foils: Results of an Intercomparison Exercise

**DOI:** 10.3390/molecules27217442

**Published:** 2022-11-02

**Authors:** Eleonora Conca, Mery Malandrino, Aleandro Diana, Ornella Abollino, Agnese Giacomino, Rafael Bartrolí, Teresa Moreno, Xavier Querol, Fulvio Amato

**Affiliations:** 1 Institute of Environmental Assessment and Water Research (IDÆA-CSIC), 08034 Barcelona, Spain; 2 Department of Chemistry, University of Turin, 10125 Turin, Italy; 3 Inter-Departmental Centre for Nanostructured Interfaces and Surfaces (NIS), University of Turin, 10125 Turin, Italy; 4 Department of Drug Science and Technology, University of Turin, 10125 Turin, Italy

**Keywords:** impactor, aluminium foils, digestion, elemental analysis, metals, atmospheric particulate matter

## Abstract

Aluminium is the most common substrate in studies using impactors for the measurement of the number or the weight of size-segregated atmospheric particulate matter (PM), as its characteristics perfectly fit impactor requirements. However, its use is not recommended by manufacturers when one of the purposes of the study is the determination of the metal content in the sample. The aim of this work was to develop an efficient analytical procedure for the removal and acid digestion of PM samples collected on aluminium foils by a cascade impactor to perform the determination of metals. The possibility of performing the trace metal analysis of PM samples collected using aluminium foils is of great importance, as it allows the determination of an accurate size distribution and the elemental composition of the PM collected on each impactor stage. Two procedures were optimised by using different digestion and analysis techniques. Both procedures were then applied to the two halves of several Dekati low-pressure impactor (DLPI) samples, and the results were critically compared. The two procedures proved to be effective in the determination of extremely low concentrations of a large suite of analytes in different size fractions of PM emitted by a brake system.

## 1. Introduction

Over the last decades, the physical and chemical characterisation of airborne particulate matter (PM) has become increasingly important due to its impact on the health of living organisms [1,2], visibility reduction [3,4], and, at a larger scale, the overall Earth climate [5,6]. Atmospheric residence times, deposition rates, and health risks are predominantly influenced by the particle size distribution. The health risks associated with PM arise from the possibility of deposition of particles in the human respiratory system; in particular, the depth that particles can travel, and hence the damages they can cause to the organism, mostly depend on the particle size [7]. For this reason, the study of the particle size distribution and of the composition of the different size fractions is extremely important. The classification of the particle sizes is often performed with regards to the aerodynamic diameter, defined as the diameter of a spherical particle of density 1 g/cm^3^ having a settling velocity equal to that of the particle in question, regardless of the actual shape of the particle [8]. Ultrafine particles (aerodynamic diameter < 0.1 µm) are potentially able to penetrate the alveolar epithelium and reach the bloodstream. Additionally, due to their large specific surface area, they are extremely reactive, a fact that often results in enhanced inflammatory potential and greater toxicity [9].

As a result of these findings, monitoring and regulation of PM have evolved, and the focus has moved from total concentrations (i.e., total suspended particulates) to smaller inhalable particles [10]. For this reason, the techniques used for sampling and collection of particles include size cut-off inlets such as impactors. Impactors are devices based on the principle that, when a flow of air sharply bends, suspended particles continue in a straight line due to their inertia. If air bending is caused by the presence of a surface, i.e., a collection plate, particles will impact on it and may stick on it. By taking into consideration that larger particles have greater inertia, it is possible to build multistage cascade impactors, in which orifice diameters become increasingly smaller at each stage, causing the air to move faster and hence the size of the impacted particles to decrease. Each collection plate will then contain particles of a characteristic size interval, which can be weighed and separately analysed [11].

To ensure high collection efficiencies and cut-points equal to the ones declared by manufacturers, the collection substrates for impactors should be thin, be nonporous, and have a smooth surface. The substrate material may vary according to measurement conditions (e.g., temperature) and chosen analytical methods; theoretically, any substrate with the above-mentioned characteristics could be used, yet published literature does not offer sufficient information on the collection efficiency of each material.

The substrate material recommended by the impactor manufacturers is aluminium foil: its thickness and the smoothness of its surface perfectly meet the requirements for collection substrates, and, in addition, it is inexpensive and easy to handle (the foils can be made of household aluminium foil). However, the hardness of the metal surfaces does not limit particle bounce, which should then be prevented by covering the surface with an appropriate sticky substance (e.g., Apiezon-L, a grease made of a combination of hydrocarbons) [12]. Furthermore, the nature of the material causes the inorganic analysis to be quite difficult to perform. Then their use is limited to gravimetric measurements, microscopy studies, and determination of organic substances [13,14,15,16]. Elemental analysis of samples collected on aluminium foils is usually performed with techniques that do not require acid digestion, such as X-ray photoelectron spectroscopy (XPS), in which aluminium interferences are removed by using an appropriate wavelength filter [17], or laser ablation techniques, in which samples are removed from the substrates by sublimation [18,19]. However, for performing analysis with techniques requiring the complete solubilisation of samples (e.g., inductively coupled plasma–optical emission spectroscopy, ICP-OES; inductively coupled plasma–mass spectrometry, ICP-MS; atomic absorption spectroscopy, AAS), substrates made of other materials, such as polycarbonate [20] and Teflon [21,22], are used. Such materials can easily be digested but do not represent the first choice from the collection efficiency point of view, as they may determine a shift of the cut-off sizes from the ones provided by the manufacturer [12,14,23]. Moreover, when using polycarbonate or Teflon filters, accurate gravimetric measurements can be quite difficult to perform, since those materials can frequently become highly electrostatic. For these reasons, the possibility of performing trace metal analysis of PM samples collected using aluminium foils is of great importance, since it allows the determination of an accurate size distribution and the elemental composition of the PM collected on each impactor stage. This possibility is crucial when analysing atmospheric particulate matter samples deriving from a braking system, as their elemental composition and size distribution are extremely characteristic and of enormous help in studying their nature, as they can provide information on the variability of the composition of different braking systems despite their low concentrations.

It is important to emphasise the lack of specific analytical studies in this area, which is the reason why this study proposed analytical solutions to broaden knowledge and to allow trace metal analysis despite the unsuitable support material.

The objective of this work was to develop an efficient analytical procedure for the removal and acid digestion of PM samples collected on aluminium foils covered with Apiezon-L by a cascade impactor in order to perform the size-resolved determination of metals in PM samples.

Two procedures were tested: one using oven digestion and the other using microwave-assisted digestion. The mixture to be used for the oven digestion was not optimised here as it had already been used in previous works [24,25,26,27]. Instead, the microwave-assisted digestion was optimised by testing five different acid mixtures. In both cases, the PM samples were removed from the aluminium foil by means of a small cotton wad, which was subsequently digested and analysed.

## 2. Results and Discussion

### 2.1. Preliminary Checks

Table 1 shows the results of testing five different acid mixtures for the microwave digestion (**Mixture A:** 4 mL HNO_3_ + 1 mL H_2_O_2_, without H_3_BO_3_; **Mixture B:** 4 mL HNO_3_ + 2 mL HF, with 0.7 g of H_3_BO_3_; **Mixture C:** 4 mL HNO_3_ + 2 mL HF, without H_3_BO_3_; **Mixture D:** 4 mL HNO_3_ + 1 mL HF + 1 mL HCl, with 0.7 g of H_3_BO_3_; and **Mixture E:** 4 mL HNO_3_ + 1 mL HF + 1 mL HCl, without H_3_BO_3_).

Overall, mixtures B and D resulted the most effective ones. It is evident that H_3_BO_3_ plays a key role in promoting the analyte recoveries, especially for the elements Ba, Ca, and Mg, probably due to the removal of insoluble fluoride compounds and the formation of more soluble BF_4_^-^ complexes [28,29]. As the results from the two mixtures including H_3_BO_3_ were similar to each other, we chose mixture B (4 mL HNO_3_ + 2 mL HF, followed by the addition of 0.7 g of H_3_BO_3_), which is simpler and ensures lower matrix blanks. Indeed, the metal concentrations in size-resolved PM samples are sometimes extremely low; for this reason, it is absolutely necessary to keep the matrix blank concentrations as low as possible.

The mixture to be used for the oven digestion was not optimised here as it had already been used in previous works [24,25,26,27].

Table 2 and Table 3 shows the results of preliminary checks for both oven digestion and microwave-assisted digestion: sample blank concentrations and relative percentage errors obtained for CRM; the relative percentage errors reported for Ca, an element not certified in the CRM NIST 1648, were estimated from the values certified for NIST 1648a (second batch of CRM “Urban particulate matter”). Considering that the standards for the analysis of the oven-digested samples were prepared in 0.1% HNO_3_, the matrix contribution (oven digestion of the acidic mixture only) had to be subtracted from the resulting concentrations. As the analysis of the microwave-digested samples was performed by using the matrix-matching approach (the standards were prepared in a matrix obtained by microwave digestion of the acidic mixture), no subtraction was performed on the resulting concentrations.

Regarding the direct digestion of aluminium foils (all supplied by the manufacturer Dekati Ltd., Kangasala, Finland), we noticed that sample blank concentrations of Al, Fe, Mn, Ni, Ti, and Zn were remarkably high with both digestion procedures. In addition, the relative percentage errors obtained for CRM, for the microwave-assisted digestion procedure, were too high for considering this as a viable option. Therefore, we decided to avoid direct digestion and focused instead on the removal of samples from their supports by using small cotton wads. Two different cotton brands were tested to choose the brand resulting in lower sample blank concentrations (the results obtained on the discarded brand are not shown). The use of cotton wads gave satisfactory results on both sample blanks and CRM, and, after leaching, the sample blank concentrations of Ca, Cr, Mg, Mn, Na, and, to a lesser extent, K further significantly decreased (Table A4). It is important to underline that, when the determination of these metals is not required, the cotton leaching process might be useless and even detrimental. Unnecessary steps in analytical procedures should always be avoided to limit the possibility of sample contamination. For this reason, the microwave-assisted digestion procedure was optimised without the cotton leaching step so as to evaluate how this choice can influence the sample results. Values reported in Table 2 as “Leached cotton on Al” represent the results of the digestion of cotton after wiping unused aluminium foils, i.e., sample blank concentrations subtracted from the final sample concentrations. When the cotton wads were used for wiping the aluminium foils, a significant increase in Fe was observed with both procedures, whereas a significant increase in Ca took place only with the oven procedure, and a significant increase in Mn was observed only with the microwave procedure.

### 2.2. Evaluation of the Method Performances

The repeatability of the analytical techniques can be considered satisfactory, as relative standard deviations (RSD—not shown) are lower than 5%, with very few exceptions. The repeatability of measurements performed with the Perkin-Elmer ICP-OES was the highest, whereas the measurements performed with the GF-AAS were the least repeatable. These values imply that, from the point of view of precision, both procedures are suitable for the analysis of airborne brake wear particles.

**Figure 1:**
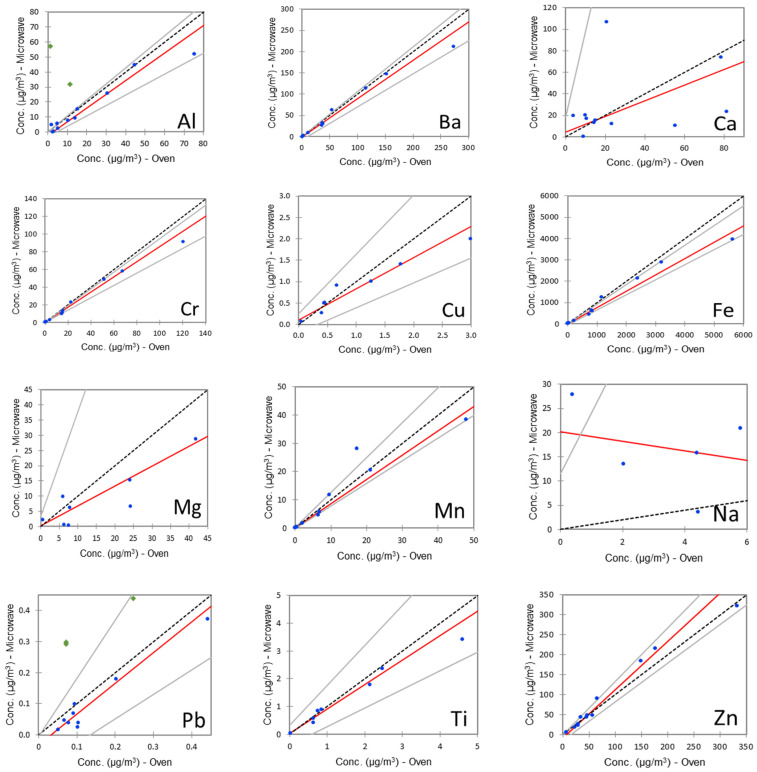
Regression models obtained with Passing–Bablok algorithm; points represented by diamonds were not included in model calculations.

The regression models obtained with the Passing–Bablok algorithm are shown in Figure 1, and the model coefficients are reported in Table 4.

For most of the analytes, the results obtained with the two techniques are in good agreement, even though we found some outliers. The latter, represented by diamond shapes in the graphs, were not included in the model calculation. The slopes are often lower than one, indicating that the microwave-assisted digestion procedure gave slightly lower results than the oven digestion procedure. The same behaviour can be found for Al, Mg, Mn, and Pb in the relative percentage errors obtained for the CRM (Table 3), indicating that, for these analytes, the oven digestion procedure provides better recoveries. For the other analytes, the sample having the highest concentration was subjected, in the oven digestion procedure, to a slight overestimate, determining a remarkable change in the slopes of the interpolation lines.

For all the analytes, the null hypothesis H_0_, stating that the relationship between the two variables is linear (slope equal to 1 and intercept equal to 0), was accepted. Nevertheless, it is important to notice that the slope and intercept confidence intervals calculated for Ca, Mg, and Na are remarkably high, demonstrating the weakness of the model built for those analytes. In addition, the slope calculated for Na is negative. The reason for the poor agreement between the results obtained for these three analytes with the two techniques might be found in the high and extremely variable content of these elements in the cotton wads used for the removal of samples from supports. Indeed, the leaching process was only performed in the oven digestion procedure, thus demonstrating the importance of this step when the determination of these metals is required. In both digestion procedures, sample blank concentrations were subtracted to sample results, but the variability of the content of Ca, Mg, and Na in cotton caused an overestimate or an underestimate of their content in some of the samples. It is important to clarify that these elements are not considered as markers for airborne brake wear particles, since their content in atmospheric PM derives from other sources (e.g., sea spray and soil dust). For the elements most commonly determined in atmospheric PM samples emitted by brake wear (e.g., Ba, Fe, Mn, and Zn), the results obtained with both procedures are generally comparable. Therefore, it seems that both methods are effective in the analysis of brake wear PM samples collected on aluminium supports, thus allowing for a single sampling for the accurate determination of both the sample weight and metal content.

Table 5 reports the analyte concentrations in cotton wads used for further wiping of blank aluminium foils and of aluminium foils after the removal of samples; the p-values resulting from the Mann–Whitney test, performed for checking the efficiency of particle removal from supports, are also reported.

The results of the test indicate that, for most of the analytes, the concentrations found by digesting the cleaned supports are not significantly higher than the concentrations of the sample blanks. The only exceptions are represented by Ba and Fe, which are among the analytes presenting the highest concentrations in the PM samples analysed; for this reason, the percentage of loss of these analytes from the samples can be considered negligible. For all the other analytes, it is possible to affirm that the removal of PM from the supports was complete.

By comparing the sample blanks of cotton wads used for wiping unused aluminium foils (Table 2, “Leached cotton on Al”) and sample blanks of cotton wads used for further wiping of blank aluminium foils (Table 5, “Cleaned blank supports”), it is evident that the latter shows a very strong release of aluminium, probably due to the previous removal of the superficial passivated film from the aluminium foil; the other analyte concentrations do not significantly vary.

### 2.3. Sample Results and Data Analysis

**Figure 2:**
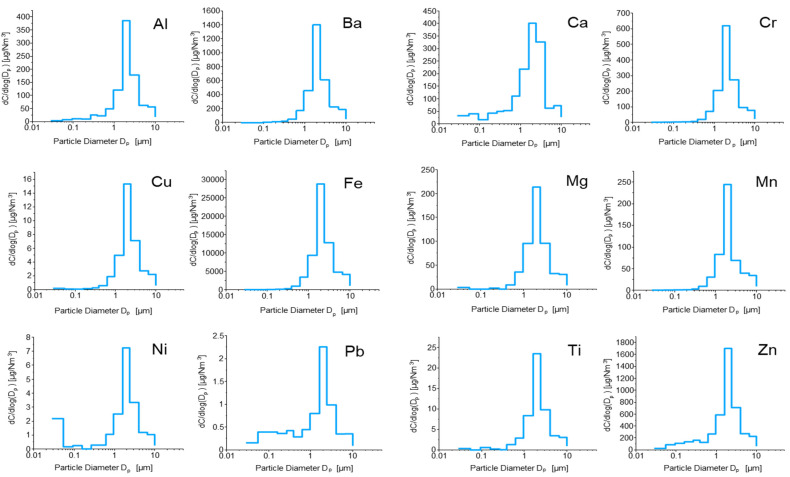
Size distribution of analytes over the 13 size fractions (oven digestion).

Figure 2 represents the size distribution of elements over the 13 size fractions analysed with the cotton-oven procedure; Cd, Co, K, and Na results could not be plotted due to the absence of a sufficient amount of data above the detection limit.

**Figure 3:**
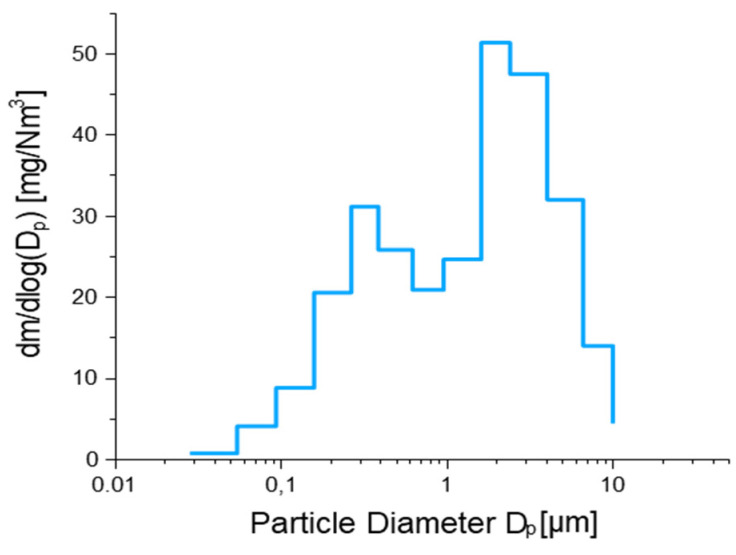
Size distribution of PM mass [mg/Nm^3^] over the 13 size fractions.

**Figure A1:**
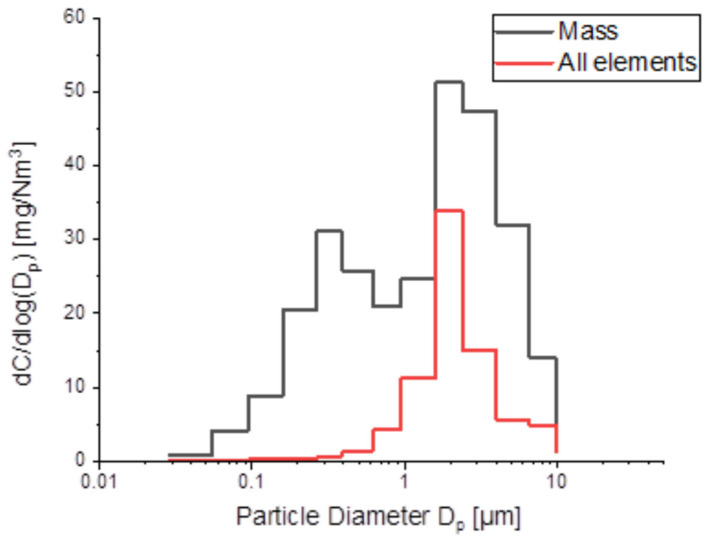
Size distribution of PM mass [(mg/Nm^3^)] (dark grey line) and of total mass explained by chemical elements determined [(mg/Nm^3^)] (red line) over the 13 size fractions.

For all the analytes, the size distribution appears unimodal, with a peak for Fraction 9 (size range 1.60 to 2.39 µm). This is coherent with the size distribution of PM weight collected for the 13 size fractions (Figure 3), even though the latter presents a bimodal distribution, with a second smaller peak for Fraction 5 (size range 0.262 to 0.382 μm). This size distribution is in line with that reported in several literature studies where various brake pads were tested [30,31,32,33]. In particular, Sanders et al. (2003) [31] considered NAO, LM, and SM types on a brake dynamometer and found particle mass size spectra with modes at 3–4 μm, despite the use of different braking scenarios. However, in tests of various brake linings including both NAO and SM types using a brake dynamometer, Garg et al. (2000) [32] reported highly varied particle mass size spectra, with a mass median diameter of 2.49 ± 3.47 μm. This variability was attributed by Sanders et al. (2003) [31], in part, to particle losses in the sampling system. We cannot exclude the fact that the differences found in the size distribution of the total mass and of the studied chemical elements (Figure A1) may be due to possible sample losses during the sampling and pre-treatment phase. Indeed, it is known that in the micron-size range, particle losses tend to increase with the particle size, and this can strongly influence the element distribution in the larger fractions. Concerning the second smaller peak for Fraction 5, it is likely that this trend may be due to carbonaceous species considering the general composition of disc and brake pads. Indeed, these particles can arise from the heat release and possible combustion of the organic materials used as binders in the brake linings, as brake temperatures rise to 550 °C during the AK Master test. However, the mass contribution of these particles is low due to their small sizes, and the mass emissions are dominated by the mechanically generated wear particles.

Table A5 shows the elemental concentrations and standard deviations found in airborne brake wear particles when the cotton-oven procedure was applied. From the latter concentrations and the PM weights reported in Table A1, the relative importance of the determined analytes was calculated. Fe represents an extremely variable and sometimes considerable portion of the PM weight, ranging from 0.5% for Fraction 4 (size range 0.157 to 0.262 µm) to 56% for Fraction 9 (size range 1.60 to 2.39 μm). Semi-metallic pads, such as the one we tested, generally contain a mixture of organic and metallic ingredients, and typically more than 50% of the pad content is represented by ferrous materials (i.e., iron powder and steel fibres). Therefore, even though one of the sources of Fe in PM emitted by brake systems might be the wear of the grey cast iron disc, it is likely that, in this case, a significant portion of the emitted Fe derives from the pad wear. The second most important element, in terms of the percentage of the emitted PM weight, is Zn, whose contribution is much more stable throughout the different size fractions: this element represents a portion ranging from 0.7% for Fraction 4 (size range 0.157 to 0.262 μm) to 3.3% for Fraction 9 (size range 1.60 to 2.39 μm). The concentrations of toxic elements, such as Cd, Ni, and Pb, are often extremely low, representing less than 0.01% of the emitted PM weight or even lying below the DL.

## 3. Materials and Methods

### 3.1. Sample Collection

The samples collected derived from airborne brake wear particles generated using a brake dynamometer, an instrumentation that simulates braking under controlled conditions, thus verifying the behaviour, the duration, and performance of the braking system. The dynamometer bench manages to simulate the mass of the vehicle to be stopped by means of a series of large flywheels on which the inertial masses correspond to the weight of the vehicle. The brake pads commercially available can be distinguished into three families according to the content of metal fibres: non-asbestos organic (NAO) pads, which present less than 10% metal fibres but a higher content of organic and/or mineral fibres; low steel (LS) pads, which present an average amount of metal fibres up to 10–30%; and semi-metallic (SM) pads, which consist of a metal fibres content ranging from 30% up to 65%. For this study, one semi-metallic brake pad was tested by means of an AK Master schedule, a test used by worldwide friction industry to screen the friction material effectiveness by varying braking parameters [34,35]; the whole dynamometer test includes 20 test sequences. The size-resolved PM samples collected for this study derive from section 9, namely “Fade 1”: 15 stops from a velocity of 100 km/h to 5 km/h were performed at a deceleration level of 0.4 g, increasing the initial temperature (T_I max_ = 550 °C). Particulate samples were collected with a Dekati low-pressure impactor (DLPI), a 13-stage cascade low-pressure impactor with cut-off sizes at 9.96, 6.60, 4.00, 2.39, 1.60, 0.949, 0.614, 0.382, 0.262, 0.157, 0.0934, 0.0549, and 0.0282 µm of particle aerodynamic diameter (reported as d_50_ in Table A1). The ‘cut-off size’ corresponds to the aerodynamic diameter of particles trapped with an efficiency of 50% on a given stage, so each size fraction collected on a cascade impactor is determined by the cut-off diameter of the current stage (lower diameter limit) and that of the previous stage (upper diameter limit). Aluminium foils (25 mm diameter) coated with Apiezon-L were employed as collection surfaces. The sample flow rate was 30 L/min.

### 3.2. Apparatus and Reagents

The dissolution of samples was carried out using alternatively a Memmert INB200 oven or a Milestone MLS-1200 Mega microwave laboratory unit. Analyses were carried out using the following instruments: an inductively coupled plasma–optical emission spectrometer (ICP-OES) and an inductively coupled plasma–mass spectrometer (ICP-MS) for the oven-digested samples, and ICP-OES and a graphite furnace atomic absorption spectrometer (GF-AAS) for the microwave-digested samples. Table A2 shows the model and features of each instrumental technique used. The choice of the suitable analysis technique for each element was evaluated depending on the sensitivity of the instrumentation and on the acid digestion mixture used to mineralise the samples. For oven digestion, the ICP-OES was used for elements present at high concentrations; the ICP-MS was selected for all those elements present at low concentrations. For microwave-assisted digestion, the ICP-OES instrument was always used for the abundant elements; the GF-AAS was used for low-concentration elements considering the matrix of the samples and the procedure that did not include an evaporation step of the solvent, which contained HF, which is not compatible with the ICP-MS instrumentation. The experimental conditions applied for ICP-OES, ICP-MS, and GF-AAS analysis are reported in Table A3. Detection limits (DLs) of the analytes of interest were experimentally determined for each technique used; DL values represent the analyte concentration corresponding to three times the standard deviation of the matrix blank (Table A3).

Reagents were all of analytical purity. Water was purified in a Milli-Q system, resulting in high purity water (HPW) with a resistivity of 18.2 MΩ∙cm. Intermediate metal standard solutions were prepared from concentrated (1000 and 10,000 mg/L) stock solutions (Sigma-Aldrich TraceCERT (St. Louis, MO, USA) or CPI International (Santa Rosa, CA, USA)) and acidified to pH = 1.5.

### 3.3. Procedures

#### 3.3.1. Oven Digestion

The oven digestion consisted of the addition of 2.5 mL HF and 1.25 mL HNO_3_ to the sample in a screw-cap Teflon vessel. The samples were then heated at 90 °C for 12 h. After cooling, 1.25 mL HClO_4_ was added and vessels were placed open on a heating plate at 240 °C for evaporating to dryness; then, 0.5 mL HNO_3_ was added and evaporated again. The solid residue was dissolved in other 0.5 mL HNO_3_, and HPW was added until the final volume of 12 mL. The absence of solid residues was ensured by centrifugation [26].

As a preliminary check, the direct digestion of aluminium foils was executed, both alone (aluminium foil only) and with 10 mg of the certified reference material (CRM) NIST 1648 (“Urban particulate matter”). Subsequently, we tried to evaluate the possibility of using small cotton wads for removing the PM samples from the aluminium foils. For this aim, 10 mg of cotton was digested, both alone (cotton only) and with 10 mg of CRM NIST 1648. Two different cotton brands were tested. Afterwards, 3 g of cotton was leached with 300 mL of HPW for 8 h by means of a rotating shaker; cotton was squeezed with a polypropylene and polyisoprene syringe and dried in an oven at 60 °C for 14 h. Cotton tests were then repeated by using 10 mg of leached cotton. Eventually, some unused aluminium foils were cleaned with 10 mg of leached cotton wetted with a drop of HNO_3_; the cotton wads were then digested for obtaining sample blank concentrations to subtract from the final sample concentrations.

After the optimisation steps, the chosen procedure was applied to one half of the 13 DLPI samples: 10 mg of leached cotton and a drop of HNO_3_ were used for wiping each aluminium foil and then digested. To verify that the removal of samples from the aluminium foils was complete, both sample blank supports and sample supports were further cleaned with 10 mg of leached cotton wetted with a drop of HNO_3_; the cotton wads were then digested, and the solutions obtained in the two cases were compared.

#### 3.3.2. Microwave-Assisted Digestion

For the microwave-assisted digestion, tetrafluoromethoxyl (TFM) vessels were used. Five different acidic mixtures were tested to find the best digesting conditions (Table 6); 30 mg of CRM BCR 176 (“City waste incinerator fly ash”) was used for this purpose.

The heating program, previously optimised for PM samples [36], consisted of six steps: 1 min at 250 W; 2 min applying no power; 5 min at 200 W; 5 min at 350 W; 5 min at 550 W; and 5 min at 250 W. When H_3_BO_3_ was added, this was carried out after the actual digestion: the vessels were previously cooled and, after the addition, a further heating step (5 min at 250 W) was appended. At the end of the treatment, the samples were diluted to 25 mL with HPW. The absence of solid residues was ensured by filtration with Whatman Grade 5 cellulose filters, previously cleaned with 20 mL HPW.

As for the oven digestion, the direct digestion of aluminium foils was executed, both alone (aluminium foil only) and with 20 mg of the CRM NIST 1648. Subsequently, 20 mg of cotton was digested, both alone (cotton only) and with 20 mg of CRM NIST 1648.

After the optimisation steps, the chosen procedure was applied to the other half of the DLPI samples: two cotton wads of 20 mg, wetted with HNO_3_, were used for wiping each aluminium foil and then digested.

### 3.4. Data Analysis

The Mann–Whitney test (one-sided, level of confidence 95%) was used for verifying whether the leaching of cotton and the wiping of the aluminium supports determined a significant increase in some analytes, and for verifying if the removal of samples from the aluminium foils was complete [37,38]. In the first two cases (leaching of cotton and wiping of aluminium supports), the test could not be performed for Cd, Co, Cu, Ni, Pb, and Zn on oven-digested samples and on Cd, Co, Cu, Ni, Pb, and Zn for microwave-digested samples, as most of the results obtained for these analytes were below the sample blank or the detection limit. In the latter case (removal of samples from supports), the test could not be performed for Cd and Co as the results obtained for these analytes were all below the detection limit. Method comparison was performed by the Passing–Bablok regression (level of confidence 95%), a nonparametric technique that does not require that one of the analytical procedures (the x variable) is exempt from error [39,40,41]. No regression model could be calculated for Cd, Co, K, and Ni due to the absence of a sufficient amount of data from the two digestion procedures. XlStat 2018.1 software package, an add-on of Microsoft Excel, was used for performing the calculations.

## 4. Conclusions

In this study, two analytical procedures for metal determination in PM collected on aluminium foils were developed, differing from each other on the digestion and analysis techniques applied. Both the procedures included the removal of samples from the supports by using small cotton wads wetted with nitric acid. The efficiency of the removal process was proved by further wiping aluminium foils after the removal of samples, whereas the efficiency of the digestion procedures was tested by means of two different CRMs. The Passing–Bablok regression was used for method comparison, and the two procedures were not significantly different at a confidence level of 95%. The two procedures proved to be effective in the determination of extremely low concentrations of a large suite of analytes in the different size fractions of the PM emitted by a braking system. However, for the accurate determination of alkali and alkaline earth metals, the choice of the cotton type and the leaching of cotton wads proved to be crucial, as they allowed a reduction of the sample blank concentrations.

## Figures and Tables

**Table 1:** Results of optimisation of microwave digestion: relative percentage errors obtained for CRM BCR 176 (“City waste incinerator fly ash”).

	Relative Percentage Errors (%)				
Analyte	Mixture A	Mixture B	Mixture C	Mixture D	Mixture E
Ba	−12	−3	−46	−1	−28
Ca	−3	9	−70	9	−61
Cd	−14	−19	−22	−21	−18
Co	−53	1	−8	−8	−14
Cr	−63	−53	−62	−70	−72
Cu	−7	3	−4	−3	3
Fe	−19	4	−29	1	1
K	−19	−1	−6	−4	3
Mg	−43	−14	−88	−6	−80
Mn	−23	−12	−23	−9	13
Na	−11	−10	−16	−5	−13
Ni	−37	−9	−5	−12	−6
Pb	−2	2	−5	−3	2
Ti	−67	−4	−5	−4	5

**Table 2:** Results of preliminary checks—sample blank concentrations.

Sample Blank Concentration (μg/L)							
Analyte	Aluminium Foil	Cotton	Leached Cotton	Leached Cotton on Al	Aluminium Foil	Cotton	Cotton on Al
	Oven	Oven	Oven	Oven	Microwave	Microwave	Microwave
Al	5 × 10^5^ ± 1 × 10^5^	34 ± 1	34 ± 9	40 ± 10	6 × 10^4^ ± 1 × 10^4^	40 ± 10	60 ± 10
Ba	<SB	0.52 ± 0.02	0.52 ± 0.05	0.46 ± 0.06	n.a.	3.1 ± 0.8	3.1 ± 0.5
Ca	<SB	310 ± 20	160 ± 30	300 ± 50	<7.6	560 ± 70	360 ± 70
Cd	<0.054	<0.054	<0.054	<0.054	<0.043	<0.043	<0.043
Co	2.0 ± 0.4	<0.018	<0.018	<0.018	0.7 ± 0.2	<0.30	<0.30
Cr	6.4 ± 0.7	0.92 ± 0.09	0.5 ± 0.2	0.5 ± 0.2	3 ± 2	<0.33	<0.33
Cu	120 ± 10	<SB	<SB	0.09 ± 0.02	24 ± 3	<0.91	<0.91
Fe	3400 ± 300	14 ± 2	13 ± 4	20 ± 3	3000 ± 700	31 ± 8	53 ± 9
K	20 ± 10	38 ± 4	20 ± 5	26 ± 4	27 ± 8	44 ± 9	110 ± 10
Mg	<SB	80 ± 20	46 ± 7	44 ± 8	<0.50	160 ± 20	140 ± 40
Mn	110 ± 10	1.07 ± 0.03	0.8 ± 0.2	0.9 ± 0.1	560 ± 70	0.8 ± 0.5	1.59 ± 0.08
Na	30 ± 20	290 ± 20	21 ± 5	30 ± 10	<30	370 ± 20	330 ± 60
Ni	22 ± 2	0.5 ± 0.1	<0.17	<0.17	13 ± 2	<1.8	<1.8
Pb	6 ± 4	0.17 ± 0.04	<SB	0.02 ± 0.01	4 ± 1	<0.18	<0.18
Ti	90 ± 20	0.9 ± 0.3	0.6 ± 0.4	0.8 ± 0.3	113 ± 7	3 ± 1	2.3 ± 0.7
Zn	120 ± 50	1.3 ± 0.7	<SB	2.1 ± 0.6	70 ± 10	4 ± 2	4 ± 2

SB = sample blank; n.a. = not analysed.

**Table 3:** Results of preliminary checks—relative percentage errors obtained for CRM NIST 1648 (“Urban particulate matter”).

	Relative Percentage Errors (%)			
Analyte	Aluminium Foil	Aluminium Foil	Leached Cotton	Cotton
	Oven	Microwave	Oven	Microwave
Al	−600	92	2	−9
Ba	−27	n.a.	−10	n.a.
Ca	−37	−78	6	−1
Cd	−1	−5	−22	−1
Co	−11	−32	−8	−31
Cr	−50	−62	−44	−38
Cu	−13	−14	−12	−12
Fe	−15	−26	−8	−9
K	−11	−29	3	−1
Mg	−12	−91	−2	−11
Mn	−25	−18	−3	−9
Na	−4	−30	5	−6
Ni	−8	9	−3	−2
Pb	−10	−20	4	−10
Ti	−15	−10	−47	−10
Zn	−31	−23	−3	−15

n.a. = not analysed; the results reported for Ca were estimated from the values certified for NIST1648a.

**Table 4:** Model coefficients obtained with Passing–Bablok regression at a confidence level of 95%.

Analyte		Value	Lower Bound	Upper Bound
**Al**	Intercept	−2.070	−2.971	0.309
	Slope	0.913	0.696	1.067
**Ba**	Intercept	−0.324	−7.572	1.012
	Slope	0.902	0.777	1.055
**Ca**	Intercept	4.363	−101.804	18.038
	Slope	0.732	−0.081	7.941
**Cr**	Intercept	0.104	−0.077	0.258
	Slope	0.859	0.699	0.949
**Cu**	Intercept	0.103	−0.196	0.255
	Slope	0.729	0.583	1.385
**Fe**	Intercept	3.438	−42.951	13.079
	Slope	0.766	0.705	0.921
**Mg**	Intercept	0.305	−23.327	3.420
	Slope	0.653	0.187	3.440
**Mn**	Intercept	0.055	−0.107	0.180
	Slope	0.861	0.799	1.238
**Na**	Intercept	20.223	−39.626	11.560
	Slope	−1.000	0.971	12.617
**Pb**	Intercept	−0.033	−0.107	−0.006
	Slope	0.993	0.792	1.878
**Ti**	Intercept	0.032	−0.398	0.327
	Slope	0.880	0.674	1.438
**Zn**	Intercept	−8.514	−15.368	−1.238
	Slope	1.210	0.972	1.344

**Table 5:** Concentrations of analytes in cotton wads used for further wiping of blank aluminium supports and of aluminium supports after removal of samples (oven digestion); *p*-values obtained with Mann–Whitney test (*p*-values lower than 0.05 are in bold) for verifying if the removal of samples from aluminium foils was complete.

Analyte	Cleaned Blank Supports (µg/kg)	Cleaned Sample Supports (µg/kg)	Mann-Whitney *p*-Values
**Al**	5000 ± 2000	6000 ± 3000	0.278
**Ba**	0.4 ± 0.1	4 ± 4	**0.005**
**Ca**	130 ± 10	130 ± 20	0.558
**Cd**	<0.054	<0.054	-
**Co**	<0.018	<0.018	-
**Cr**	0.7 ± 0.3	1.3 ± 0.7	0.127
**Cu**	0.7 ± 0.4	<SB	-
**Fe**	11 ± 2	130 ± 80	**0.018**
**K**	4 ± 3	<SB	-
**Mg**	50 ± 9	40 ± 6	0.980
**Mn**	1.5 ± 0.6	3 ± 1	0.070
**Na**	60 ± 40	30 ± 20	0.821
**Ni**	<0.17	<0.17	-
**Pb**	0.2 ± 0.2	0.2 ± 0.1	0.278
**Ti**	0.3 ± 0.1	1.3 ± 0.4	**0.022**
**Zn**	2 ± 2	5 ± 2	0.232

**Table 6:** Acid mixtures tested for microwave-assisted digestion.

Mixture	Acids	H_3_BO_3_
A	4 mL HNO_3_ + 1 mL H_2_O_2_	No
B	4 mL HNO_3_ + 2 mL HF	0.7 g
C	4 mL HNO_3_ + 2 mL HF	No
D	4 mL HNO_3_ + 1 mL HF + 1 mL HCl	0.7 g
E	4 mL HNO_3_ + 1 mL HF + 1 mL HCl	No

## Data Availability

Data, associated metadata, and calculation tools are available from the corresponding author (mery.malandrino@unito.it).

## Appendix A

**Table A1:** Cut-off diameter (d_50_) and collected weight of each size fraction.

Fraction	d_50_ (µm)	Mass (mg/Nm^3^)
1	0.0282	0.235
2	0.0549	1.076
3	0.0934	2.273
4	0.157	5.151
5	0.262	5.693
6	0.382	5.920
7	0.614	4.405
8	0.949	6.239
9	1.60	10.03
10	2.39	11.85
11	4.00	7.780
12	6.60	2.807
13	9.96	3.496

**Table A2:** Instrumental techniques used for analysis of oven-digested and microwave-digested samples.

	Technique	Model	Features	Analytes
Oven digestion	ICP-OES	Thermo Fisher Scientific iCAP 6500 Radial	MicroFlow PFA-ST nebuliser, cyclonic spray chamber, Échelle monochromator, CID detector for simultaneous analysis	Al, Ca, Fe, K, Mg, Na
	ICP-MS	Thermo Fisher Scientific X-Series II	Meinhard nebuliser, Peltier-cooled conical spray chamber, quadrupole analyser, discrete dynode electron multiplier	Ba, Cd, Co, Cr, Cu, Mn, Ni, Pb, Ti, Zn
Microwave digestion	ICP-OES	Perkin Elmer Optima 7000 DV	Mira Mist nebuliser, cyclonic spray chamber, dual Échelle monochromator, dual CCD detector	Al, Ba, Ca, Fe, K, Mg, Mn, Na, Ti, Zn
	GF-AAS	Perkin Elmer 5100	Zeeman-effect background correction, HGA 600 graphite furnace	Cd, Co, Cr, Cu, Ni, Pb

**Table A3:** Experimental conditions applied for ICP-OES, ICP-MS, and GF-AAS analysis and relative detection limits (DLs).

ICP-OES				
Analyte	Λ (nm)		Torch Position	DL (μg/L)
Al	167.0		Radial	30
Ca	317.9		Radial	300
Fe	259.9		Radial	14
K	766.4		Radial	150
Mg	279.5		Radial	42
Na	589.5		Radial	170
Al	396.1		Axial	2.7
Ba	233.5		Axial	1.3
Ca	317.9		Radial	7.6
Fe	238.2		Axial	1.0
K	766.5		Radial	1.8
Mg	280.3		Radial	0.50
Mn	257.6		Axial	0.79
Na	589.6		Radial	30
Ti	334.9		Axial	0.2
Zn	206.2		Axial	3.3
**ICP-MS**				
**Analyte**	**Isotope**			**DL (** **μg/L)**
Ba	138			0.13
Cd	111			0.054
Co	59			0.018
Cr	52			3.0
Cu	65			0.40
Mn	55			0.40
Ni	60			0.17
Pb	208			1.6
Ti	47			2.3
Zn	64			7.3
**GF-AAS**				
**Analyte**	**λ** **(nm)**	**T Roast.** **(°C)**	**T Atom.** **(°C)**	**DL** **(** **μg/L)**
Cd	228.8	500	1500	0.043
Co	240.7	1400	2400	0.30
Cr	357.9	1650	2500	0.33
Cu	324.8	1200	2000	0.91
Ni	232.0	1400	1800	1.8
Pb	283.3	850	1800	0.18

**Table A4:** *p*-values obtained with Mann–Whitney test (*p*-values lower than 0.05 are in bold) for verifying if the leaching of cotton and the wiping of aluminium supports determined a significant increase in some analytes.

	Mann–Whitney *p*-Values		
Analyte	Cotton Leaching	Al Wiping	Al Wiping
	Oven	Oven	Microwave
Al	0.298	0.228	0.100
Ba	0.449	0.967	0.719
Ca	**0.008**	**0.001**	1.000
Cr	**0.047**	0.392	-
Fe	0.289	**0.012**	**0.048**
K	0.067	0.124	-
Mg	**0.006**	0.721	0.834
Mn	**0.005**	0.298	**0.048**
Na	**0.011**	0.197	0.912
Ti	0.178	0.222	0.807
Zn	-	-	0.408

**Table A5:** Elemental concentrations and standard deviations found in airborne brake wear particles (oven procedure).

Analyte (conc)		F1	F2	F3	F4	F5	F6	F7
**Al**	(µg/Nm^3^)	1.28 ± 0.03	1.976 ± 0.003	2.829 ± 0.001	2.59 ± 0.05	4.69 ± 0.08	5.20 ± 0.08	10.45 ± 0.09
**Ba**	(µg/Nm^3^)	0.156 ± 0.002	0.431 ± 0.005	0.60 ± 0.01	1.239 ± 0.007	2.46 ± 0.04	10.66 ± 0.04	35.6 ± 0.1
**Ca**	(µg/Nm^3^)	10.0 ± 0.1	10.56 ± 0.04	4.01 ± 0.02	10.54 ± 0.05	9.07 ± 0.04	11.98 ± 0.02	23.2 ± 0.1
**Cd**	(ng/Nm^3^)	<3.7	<3.8	<3.9	<4.0	<3.8	<3.6	<4.2
**Co**	(ng/Nm^3^)	<SB	<SB	<SB	<SB	<SB	<SB	39.4 ± 0.1
**Cr**	(µg/Nm^3^)	0.0611 ± 0.0009	0.138 ± 0.002	0.272 ± 0.005	0.445 ± 0.002	0.96 ± 0.01	4.24 ± 0.02	14.8 ± 0.1
**Cu**	(ng/Nm^3^)	53 ± 3	19.0 ± 0.9	12.2 ± 0.3	34.8 ± 0.5	43 ± 1	128 ± 9	396 ± 7
**Fe**	(µg/Nm^3^)	4.0 ± 0.1	9.40 ± 0.04	14.4 ± 0.1	26.4 ± 0.1	48.6 ± 0.4	221 ± 2	723 ± 7
**K**	(µg/Nm^3^)	8.8 ± 0.4	<7.3	<7.6	<7.8	<7.3	<7.0	<8.1
**Mg**	(ng/Nm^3^)	1100 ± 10	<SB	<SB	553.9 ± 0.4	18.63 ± 0.05	2070 ± 30	7560 ± 80
**Mn**	(µg/Nm^3^)	0.0224 ± 0.0006	0.081 ± 0.002	0.116 ± 0.004	0.240 ± 0.006	0.490 ± 0.006	2.021 ± 0.008	6.39 ± 0.02
**Na**	(µg/Nm^3^)	2.04 ± 0.01	<SB	<SB	<SB	<SB	<SB	4.4 ± 0.2
**Ni**	(ng/Nm^3^)	690 ± 10	41 ± 2	59 ± 4	< 13	51 ± 2	62 ± 3	220 ± 10
**Pb**	(ng/Nm^3^)	50 ± 1	104 ± 3	102 ± 2	91.6 ± 0.6	78 ± 1	66.2 ± 0.1	94.9 ± 0.9
**Ti**	(ng/Nm^3^)	114 ± 2	<SB	155 ± 7	59 ± 3	15.7 ± 0.9	310 ± 10	620 ± 20
**Zn**	(µg/Nm^3^)	7.11 ± 0.05	22.82 ± 0.04	28.6 ± 0.4	34.08 ± 0.09	29.5 ± 0.3	29.08 ± 0.07	56.1 ± 0.4
**Analyte (conc)**		**F8**	**F9**	**F10**	**F11**	**F12**	**F13**	
**Al**	(µg/Nm^3^)	30.7 ± 0.7	75.4 ± 0.8	44.5 ± 0.6	15.2 ± 0.3	11.34 ± 0.06	14.02 ± 0.07	
**Ba**	(µg/Nm^3^)	115 ± 1	274 ± 1	152 ± 1	53.5 ± 0.4	37.39 ± 0.08	37.2 ± 0.1	
**Ca**	(µg/Nm^3^)	55.2 ± 0.3	78.326 ± 0.001	81.4 ± 0.1	15.04 ± 0.03	14.48 ± 0.06	20.9 ± 0.3	
**Cd**	(ng/Nm^3^)	<3.9	<3.6	<3.8	<3.5	<4.0	<3.1	
**Co**	(ng/Nm^3^)	161 ± 4	384 ± 7	218 ± 9	73 ± 3	42 ± 4	45.3 ± 0.4	
**Cr**	(µg/Nm^3^)	51.5 ± 0.6	120.6 ± 0.7	67.8 ± 0.7	22.8 ± 0.2	15.4 ± 0.1	15.64 ± 0.08	
**Cu**	(ng/Nm^3^)	1260 ± 20	2990 ± 20	1770 ± 20	660 ± 30	440 ± 20	451 ± 4	
**Fe**	(µg/Nm^3^)	2375 ± 2	5630 ± 20	3200 ± 10	1169 ± 1	840 ± 9	823 ± 4	
**K**	(µg/Nm^3^)	<7.6	<7.1	<7.4	<6.9	<7.8	<6.0	
**Mg**	(ng/Nm^3^)	24270 ± 50	41800 ± 20	24000 ± 200	7897 ± 5	6234 ± 2	5990 ± 20	
**Mn**	(µg/Nm^3^)	21.0 ± 0.2	47.9 ± 0.3	17.2 ± 0.3	9.6 ± 0.2	6.86 ± 0.05	6.84 ± 0.06	
**Na**	(µg/Nm^3^)	9.6 ± 0.2	5.79 ± 0.02	4.42 ± 0.03	<SB	<SB	0.383 ± 0.001	
**Ni**	(ng/Nm^3^)	640 ± 10	1410 ± 50	830 ± 20	287 ± 5	207 ± 2	190 ± 20	
**Pb**	(ng/Mm^3^)	203 ± 3	441 ± 4	247 ± 2	86.7 ± 0.3	72 ± 1	71.8 ± 0.4	
**Ti**	(ng/Nm^3^)	2130 ± 80	4600 ± 30	2460 ± 30	850 ± 30	620 ± 20	750 ± 10	
**Zn**	(µg/Nm^3^)	148.9 ± 0.8	332.5 ± 0.3	177 ± 2	65.5 ± 0.6	45.4 ± 0.2	46.6 ± 0.2	

## References

[1] Mukhtar A., Limbeck A. (2013). Recent developments in assessment of bio-accessible trace metal fractions in airborne particulate matter: A review. Anal. Chim. Acta.

[2] Pelfrene A., Cave M.R., Wragg J., Douay F. (2017). In Vitro Investigations of Human Bioaccessibility from Reference Materials Using Simulated Lung Fluids. Int. J. Environ. Res. Public Health.

[3] Zhao H., Che H., Zhang X., Ma Y., Wang Y., Wang H., Wang Y. (2013). Characteristics of visibility and particulate matter (PM) in an urban area of Northeast China. Atmos. Pollut. Res..

[4] Zou J., Liu Z., Hu B., Huang X., Wen T., Ji D., Liu J., Yang Y., Yao Q., Wang Y. (2018). Aerosol chemical compositions in the North China Plain and the impact on the visibility in Beijing and Tianjin. Atmos. Res..

[5] Finlayson-Pitts B.J., Pitts J.N. (1999). Chapter 14: Global Tropospheric Chemistry and Climate Change. Climate Change, Chemistry of the Upper and Lower Atmosphere.

[6] Giardi F., Becagli S., Traversi R., Frosini D., Severi M., Caiazzo L., Ancillotti C., Cappelletti D., Moroni B., Grotti M. (2016). Size distribution and ion composition of aerosol collected at Ny-Ålesund in the spring–summer field campaign 2013. Rend. Lincei-Sci. Fis..

[7] Asgharian B., Hofmann W., Bergmann R. (2001). Particle Deposition in a Multiple-Path Model of the Human Lung. Aerosol Sci. Technol..

[8] Sullivan R.C., Prather K.A. (2005). Recent Advances in Our Understanding of Atmospheric Chemistry and Climate Made Possible by On-Line Aerosol Analysis Instrumentation. Anal. Chem..

[9] Romanazzi V., Casazza M., Malandrino M., Maurino V., Piano A., Schilirò T., Gilli G. (2014). PM10 size distribution of metals and environmental-sanitary risk analysis in the city of Torino. Chemosphere.

[10] Elmes M., Gasparon M. (2017). Sampling and single particle analysis for the chemical characterisation of fine atmospheric particulates: A review. J. Environ. Manag..

[11] Finlayson-Pitts B.J., Pitts J.N. (1999). Chapter 11: Analytical Methods and Typical Atmospheric Concentrations for Gases and Particles. Climate change, Chemistry of the Upper and Lower Atmosphere.

[12] Dekati Ltd. (2016). Substrates and Filters for Dekati® Impactors (Vers 6.3), Dekati® Accessory.

[13] Kleeman M.J., Schauer J.J., Cass G.R. (1999). Size and Composition Distribution of Fine Particulate Matter Emitted from Wood Burning, Meat Charbroiling, and Cigarettes. Environ. Sci. Technol..

[14] Noel A., L’Esperance G., Cloutier Y., Plamondon P., Boucher J., Philippe S., Dion C., Truchon G., Zayed J. (2013). Assessment of the contribution of electron microscopy to nanoparticle characterization sampled with two cascade impactors. J. Occup. Environ. Hyg..

[15] Pakkanen T.A., Kerminen V.M., Loukkola K., Hillamo R.E., Aarnio P., Koskentalo T., Maenhaut W. (2003). Size distributions of mass and chemical components in street-level and rooftop PM1 particles in Helsinki. Atmos. Environ..

[16] Scheinhardt S., Spindler G., Leise S., Müller K., Iinuma Y., Zimmermann F., Matschullat J., Herrmann H. (2013). Comprehensive chemical characterisation of size-segregated PM10 in Dresden and estimation of changes due to global warming. Atmos. Environ..

[17] Carabali G., Castro T., De La Cruz W., Peralta O., Varela A., Amelines O., Rivera M., Ruiz-Suarez G., Torres-Jardón R., Martines-Quiroz E. (2016). Morphological and chemical characterization of soot emitted during flaming combustion stage of native-wood species used for cooking process in western Mexico. J. Aerosol Sci..

[18] Gligorovski S., Van Elteren J.T., Grgic I. (2008). A multi-element mapping approach for size-segregated atmospheric particles using laser ablation ICP-MS combined with image analysis. Sci. Total Environ..

[19] Xue J., Li Y., Xie X., Xiong C., Liu H., Chen S., Nie Z., Chen C., Zhao J. (2017). Characterization of organic aerosol in Beijing by laser desorption ionization coupled with Fourier Transform Ion Cyclotron Resonance Mass spectrometry. Atmos. Environ..

[20] Gälli Purghart B.C., Nyffeler U.P., Schindler P.W. (1990). Metals in airborne particulate matter in rural Switzerland. Atmos. Environ..

[21] Hsieh Y.K., Chen L.K., Hsieh H.F., Huang C.H., Wang C.F. (2011). Elemental analysis of airborne particulate matter using an electrical low-pressure impactor and laser ablation/inductively coupled plasma mass spectrometry. J. Anal. Atom. Spectrom..

[22] Tan J., Duan J., Zhen N., He K., Hao J. (2016). Chemical characteristics and source of size-fractionated atmospheric particle in haze episode in Beijing. Atmos. Res..

[23] van Gulijk C., Marijnissen J.C.M., Makkee M., Moulijn J.A. (2003). Oil-soaked sintered impactors for the ELPI in diesel particulate measurements. J. Aerosol Sci..

[24] Querol X., Alastuey A., Rodriguez S., Plana F., Mantilla E., Ruiz C.R. (2001). Monitoring of PM10 and PM2.5 around primary par-ticulate anthropogenic emission sources. Atmos. Environ..

[25] Vicente E.D., Duarte M.A., Tarelho L.A.C., Nunes T.F., Amato F., Querol X., Colombi C., Gianelle V., Alves C.A. (2015). Particulate and gaseous emissions from the combustion of different biofuels in a pellet stove. Atmos. Environ..

[26] Minguillón M.C., Campos A.A., Cárdenas B., Blanco S., Molina L.T., Querol X. (2014). Mass concentration, composition and sources of fine and coarse particulate matter in Tijuana, Mexico, during Cal-Mex campaign. Atmos. Environ..

[27] Pay M.T., Jiménez-Guerrero P., Jorba O., Basart S., Querol X., Pandolfi M., Baldasano J.M. (2012). Spatio-temporal variability of concentrations and speciation of particulate matter across Spain in the CALIOPE modeling system. Atmos. Environ..

[28] Aldabe J., Santamaría C., Elustondo D., Lasheras E., Santamaría J.M. (2013). Application of microwave digestion and ICP-MS to simultaneous analysis of major and trace elements in aerosol samples collected on quartz filters. Anal. Methods.

[29] Wilson M.A., Burt R., Lee C.W. (2006). Improved Elemental Recoveries in Soils with Heating Boric Acid Following Microwave Total Digestion. Commun. Soil Sci. Plan..

[30] Harrison R.M., Allan J., Carruthers D., Heal M.R., Lewis A.C., Marner B., Murrells T., Williams A. (2021). Non-exhaust vehicle emissions of particulate matter and VOC from road traffic: A review. Atmos. Environ..

[31] Sanders P.G., Xu N., Dalka T.M., Maricq M.M. (2003). Airborne brake wear debris: Size distributions, composition, and a comparison of dynamometer and vehicle tests. Environ. Sci. Technol..

[32] Garg B.D., Cadle S.H., Mulawa P.A., Groblicki P.J., Laroo C., Parr G.A. (2000). Brake wear particulate matter emissions. Environ. Sci. Technol..

[33] Wahlstrom J., Söderberg A., Olander L., Jansson A., Olofsson U. (2010). A pin-on-disc simulation of airborne wear particles from disc brakes. Wear.

[34] Aranganathan N., Bijwe J. (2016). Development of copper-free eco-friendly brake-friction material using novel ingredients. Wear.

[35] (2014). Dynamometer Global Brake Effectiveness, Surface Vehicle Recommended Practice.

[36] Malandrino M., Casazza M., Abollino O., Minero C., Maurino V. (2016). Size resolved metal distribution in the PM matter of the city of Turin (Italy). Chemosphere.

[37] Mann H.B., Whitney D.R. (1947). On a test of whether one of two random variables is stochastically larger than the other. Ann. Math. Stat..

[38] Sheppard P.R., Helsel D.R., Speakman R.J., Ridenour G., Witten M.L. (2012). Additional analysis of dendrochemical data of Fallon, Nevada. Chem-Biol. Interact..

[39] Passing H., Bablok W. (1983). A new biometrical procedure for testing the equality of measurements from two different analytical methods. Application of linear regression procedures for method comparison studies in clinical chemistry, Part 1. J. Clin. Chem. Clin. Biochem..

[40] Malegori C., Nascimento Marques E.J., de Freitas S.T., Pimentel M.F., Pasquini C., Casiraghi E. (2017). Comparing the analytical performances of Micro-NIR and FT-NIR spectrometers in the evaluation of acerola fruit quality, using PLS and SVM regression algorithms. Talanta.

[41] Arendse E., Fawole O.A., Magwaza L.S., Nieuwoudt H., Opara U.L. (2018). Comparing the analytical performance of near and mid infrared spectrometers for evaluating pomegranate juice quality. LWT.

